# Ultra depth of field microscopy: a novel method for observing and characterizing of articular cartilage surface

**DOI:** 10.1186/s12967-026-08060-x

**Published:** 2026-03-25

**Authors:** Guohao Zhang, Ningning Ma, Jihao Wen, Tao Zhang, Ruxing Liu, Mengyu Fu, Li Guo, Xiaochun Wei

**Affiliations:** Department of Orthopedics, Shanxi Key Laboratory of Bone and Soft Tissue Injury Repair, Second Hospital of Shanxi Medical University, Taiyuan, Shanxi 030001 PR China

**Keywords:** Ultra depth of field microscopy, Articular cartilage, Three-dimensional characterization, Superfical zone damage detection

## Abstract

**Background:**

The surface microstructures of cartilage are closely related to its functions. Accurate observation of cartilage surface is key to researching cartilage-related diseases. Traditional observation methods have limitations: conventional optical microscopes have a narrow depth of field and cannot clearly image the uneven cartilage surface; other methods either have low resolution or damage the sample. Ultra depth of field microscopy(UDFM)is a new high-precision method for cartilage surface research. It can non-invasively show clear 3D microstructural details of the cartilage surface without damaging the sample. At present, its application in cartilage surface research is still in the exploration stage, and it is of great significance to use this new method for related research.

**Methods:**

Fresh porcine knee joints and discarded cartilage tissue from human total knee arthroplasty were utilized to prepare a range of cartilage damage models and sectioned specimens. UDFM was employed to examine the cartilage surface, cross-sections, and lateral profiles at various magnifications (10–2000×), generating both two-dimensional and three-dimensional topographic images. The integrated UDFM software was used for qualitative assessment of surface morphology, cellular architecture, and damage characteristics, as well as for quantitative measurements of height differences, surface roughness, fissure length, and stained area.

**Results:**

UDFM provides non-destructive and direct visualization of the authentic three-dimensional morphology and subtle surface damage of articular cartilage, with 3D topographic mapping precisely delineating the depth and extent of lesions. This technology enables continuous, high-resolution observation of the macroscopic appearance, surface microarchitecture, and cellular morphology of cartilage across a broad range of magnifications. In fresh specimens, it reliably reveals in situ paired or clustered chondrocytes. The OPt-SEM special lighting mode further enhances the detection sensitivity for subtle surface damage. UDFM delivers rapid and highly accurate quantitative measurements of surface height differences, roughness, fissure length, and stained area, all with excellent reproducibility. In comparison to conventional techniques, UDFM is straightforward to operate, eliminates the need for complex sample preparation, and preserves the native structure of cartilage, thereby effectively addressing the inherent limitations of stereomicroscopy and scanning electron microscopy.

**Conclusion:**

Ultra depth of field microscopy is an efficient, non-destructive, and precise tool for three-dimensional observation of cartilage surfaces, offering high resolution, large depth of field, convenient operation, and strong quantitative capabilities. This technology can comprehensively and authentically reflect the microstructure and damage characteristics of cartilage surfaces, providing a new technical approach and objective quantitative method for basic and clinical cartilage research, early disease diagnosis, and evaluation of repair efficacy, with broad application prospects.

**Supplementary Information:**

The online version contains supplementary material available at 10.1186/s12967-026-08060-x.

## Introduction

Articular cartilage is a highly differentiated tissue covering the ends of bones, characterized by a smooth surface and unique biomechanical properties. Its primary functions include cushioning joint loads, dispersing stress, maintaining joint homeostasis, and providing lubrication [1–2]. The cartilage surface, as the direct interface with the external environment, plays critical roles in material exchange [3], stress distribution [4], and lubrication [5]. Subtle alterations in cartilage surface microstructure are early signs of degenerative joint diseases [6–8], and surface morphology and integrity directly determine cartilage mechanical performance and biological function [9–11]. Therefore, accurate and non-destructive characterization of the three-dimensional surface microstructure is of great significance for articular cartilage research.

However, currently available techniques for cartilage surface observation have notable limitations. Conventional optical stereomicroscopy suffers from restricted magnification, shallow depth of field, and limited observation range, making it difficult to obtain comprehensive three-dimensional morphological information. Scanning electron microscopy (SEM) provides ultra-high resolution, but requires complex, destructive sample preparation including dehydration and coating, which distorts the native physiological structure of cartilage. Currently, there remains a lack of reliable imaging methods that can fill the gap between low and ultra-high magnification.

UDFM is an innovative optical imaging technique that combines advanced optical design and depth-of-field extension algorithms. It allows automatic acquisition and intelligent fusion of multi-focal plane images in one exposure, generating high-resolution full-field two-dimensional images and three-dimensional topographic reconstructions. UDFM requires no complex sample preparation and offers the advantages of non-invasiveness, simple operation, and high imaging speed, and has been applied in materials science, electronic engineering, and biomedical research [12–14]. However, its utility for characterizing articular cartilage surfaces has not been systematically evaluated.

This study systematically explores the application of UDFM in articular cartilage surface characterization, aiming to provide an efficient, non-destructive, and high-precision imaging tool for basic and clinical cartilage research.

### UDFM equipment (KEYENCE, VHX-X1, Japan)

#### Components and functions

- UDFM Main Console Display: Includes system algorithm control, digital signal analysis and processing, as well as coordinated control of the electromechanical unit.
- Electromechanical Unit: Comprises a motorized Z-axis stand, XY-axis motorized stage, camera, illumination unit, etc. This unit enables two-dimensional and three-dimensional field movement and allows adjustment of the light source mode.
- Optical Magnification Lens: Magnifies the target sample through an optical lens assembly, with magnification ranging from 20× to 2000×.
- Digital Camera Lens: Equipped with a CMOS sensor, it converts optical signals into digital signals and allows real-time image capture.
- Control Handle: Includes a joystick and operation buttons, providing convenient operation and integrated control. (Supplementary Fig. 1)

#### Working principle

The core principle of UDFM is to break through the depth-of-field limitation of traditional optical microscopes by means of high-precision Z-axis movement and intelligent fusion of multiple images from different focal planes, thereby obtaining full-field clear images and reconstructing three-dimensional morphologies in one go.The device optically magnifies the target area using an optical lens, transmitting the magnified optical signal to the CMOS (Complementary Metal-Oxide-Semiconductor) image sensor within the camera unit. The camera unit then converts the received optical signal into a digital signal, which is transmitted to the UDFM main console (Supplementary Fig. 2).

The UDFM main console processes the digital signal and displays the resulting image on the screen, while also performing image optimization. Quantitative analysis can be conducted within the console, including two-dimensional linear measurements, area measurements, and automatic identification of specific image features based on brightness or color differences. Additionally, the UDFM main console can actively control the connected motorized stand, adjusting the vertical movement of the motor and the horizontal movement of the stage. These functions enable 3D depth-of-field synthesis and image stitching (Supplementary Fig. 3).

#### Principle of 3D depth-of-field synthesis

Optical imaging systems are constrained by a fixed focal plane at a given magnification, and only regions matching the focal distance appear sharp. For uneven or three-dimensional samples, only focal-plane areas are in focus, while regions outside this plane appear blurred. UDFM overcomes this limitation by using a motorized Z-axis to adjust the lens position vertically, sequentially bringing different heights of the sample into focus. The system acquires fully focused images at multiple Z-positions and records corresponding height information, then reconstructs these data into a fully focused three-dimensional topography covering the entire field of view.

For high-magnification imaging, the broad spectrum of white light (400–700 nm) may introduce optical interference. To mitigate this effect, polarized lens components and DIC (Differential Interference Contrast) prisms can be applied to enhance contrast and reduce interference.

#### Basic operating procedure

- The specimen was placed upright on the UDFM stage, and the lens was adjusted to be perpendicular to the cartilage surface. Observations were initiated using a 20× objective lens with the light source set to “edge-center enhancement” to locate the region of interest. The magnification was then increased to the desired level as needed.
- For depth-of-field imaging, the lower boundary was defined by adjusting the lens to a blurred position. The “Depth-of-Field Capture” function was activated, and the lens was automatically moved upward until the image became blurred again to determine the upper boundary. This procedure acquired a sequence of fully focused images across the entire measured depth range.

#### Observable parameters of the articular cartilage surface and specific operating procedures

##### Qualitative analysis

a. Depth-of-field images at different magnifications:

The observed features vary depending on the magnification and can be selected as needed.

*Method: Gradually increase the magnification to 100×*,* 200×*,* 400×*,* and 2000× to observe the surface morphology of cartilage at different scales.*

b. Surface morphology and 3D reconstruction images:

These allow for direct visualization and measurement of changes in cartilage surface morphology.

*Method: Perform 3D image processing on the depth-of-field images*,* adjusting the 3D height parameter according to the surface undulation (set to “height 100” in this study). Use the “leveling” function to correct for any height differences caused by uneven specimen placement. Click “Generate 3D Image” to obtain a three-dimensional topographic map from the depth-of-field images.*

c. OPt-SEM:

OPt-SEM is a dedicated imaging mode that enhances surface contrast through unilateral illumination.UDFM’s unique illumination system enables the detection of subtle surface damage by varying the direction of the light source.

*Method: At 50× magnification*,* press the “OPt-SEM” detection button on the control handle to perform OPt-SEM imaging under a single light source.*

##### Quantitative analysis

a. Height difference:

Measures the height difference between any two selected points.

b. Length/distance:

Measures the linear distance between any two selected points.

*Method: Using a depth-of-field image captured at 50× magnification*,* identify the cartilage defect area to be measured. Use the “Measurement” function to select “Height between two points” and “Distance between two points” for regional height and length measurements. Repeat each measurement three times and calculate the average.*

c. Surface roughness:

Measures the roughness value of the target area, reflecting the surface undulation of the specimen.

*Method: Select three regions within the area of interest*,* increase the magnification to 400×*,* and repeat the depth-of-field imaging process. Use the “Roughness Measurement” function to select the entire field of view for roughness assessment. Average the roughness values from the three regions to obtain the roughness value for that area.*

d. Colored area and proportion measurement:

UDFM features a unique color recognition system that can measure the area of regions with color differences and calculate their proportion relative to the total area.

*Method*:

*a. Select the observation area: Use the software to click “Extract Area” and outline the region of interest.*

*b. Capture the target color region: Click “Capture Color” and move to the color region to be measured*,* setting the color target.*

*c. Click “Calculate” to obtain the area and proportion of the captured color region within the selected area.*

#### Reproducibility assessment

All quantitative data will be re-evaluated one week after measurement by examiner A. The depth-of-field images will be assessed again by another independent examiner B, using the same method. The differences between the two sets of measurements will be compared. Both A and B are independent of the study.

### Specimen materials and preparation methods

#### Source of cartilage tissue specimens

a. Porcine knee joints:

Adult, 6-month-old male Landrace pigs were selected. Within 8 h post-slaughter (from a local abattoir), knee joints were harvested, ensuring the joint capsule remained intact and undamaged. The skin, fascia, and joint capsule were sequentially incised according to anatomical layers, and the anterior/posterior cruciate ligaments and medial collateral ligament were severed to expose the cartilage. Cartilage with a bright color and smooth surface was selected. Using a hollow cartilage drill, cylindrical cartilage plugs (8 mm in diameter, 10 mm in height) were extracted from the femoral trochlea and stored in PBS solution at 4 °C for later use. (*n* = 3, with three specimens per group derived from three distinct individuals).

b. Human cartilage tissue:

Discarded cartilage tissue from total knee arthroplasty (TKA) procedures at the Second Hospital of Shanxi Medical University was also collected as specimen material. (*n* = 3, with three specimens per group derived from three distinct individuals).

(Ethics approval number: [2024] YX No. 307)

#### Specimen processing

a. Surface observation:

Remove surface moisture or synovial fluid from the cartilage tissue using clean absorbent paper to keep the specimen dry and clean. This helps to avoid refraction or reflection artifacts during imaging.

b. Section (cross-sectional) observation:

*Vibratome sectioning method*: Place the surface of the cartilage plug on a vibratome. Set the vibratome parameters to a depth of 10 μm and a speed of 2 mm/s to obtain a cross-sectional surface of the cartilage, allowing for clear observation of the section structure.

c.Lateral (side profile) observation:

*Vibratome sectioning method*: Place the side of the cartilage plug on the vibratome. Set the parameters to a depth of 10 μm and a speed of 2 mm/s to obtain 10 μm-thick slices. Place the sliced sections onto glass slides for clear observation of the cartilage side profile.

*Liquid nitrogen freeze-fracture method*: Place the cartilage plug in liquid nitrogen. While in the frozen state, make a small incision with a scalpel, then completely fracture the cartilage plug along the incision to expose the lateral profile of the cartilage.

#### Specimen observation conditions

- To maintain specimen freshness, cartilage tissue should be used within 8 h after excision to allow for clear observation of chondrocyte structures and to prevent structural shrinkage or disintegration.
- The specimen size should not exceed the maximum movement range of the platform’s XY axes (maximum 8 cm in the X direction and 8 cm in the Y direction).
- The optimal environmental conditions for observation are a temperature of 26–28 °C and humidity of 50%–60%. The environment should be clean and dry, and the specimen surface should be free of any liquid.
- The observation procedure should be completed within 5–10 min to avoid specimen drying and cell death, which could affect the results.
- For cellular observation, fresh specimens with a post-excision time of less than eight hours should be selected.

#### Special handling of cartilage

##### Preparation of cartilage injury models

To clearly demonstrate the ability of UDFM to accurately reflect the characteristics of damaged cartilage, the cartilage surface was subjected to friction treatment to create injury models.Fine sandpaper (200 grit) was used to rub the cartilage surface.Controlled forces of 1 N, 2 N, and 3 N were applied to establish mild, moderate, and severe cartilage injury models, respectively. All procedures were performed by the same operator to ensure consistency.

##### India ink staining

The damaged cartilage was immersed in India ink solution for 5 min, then rinsed for 5 min until the color no longer faded. The surface moisture was removed with absorbent paper, and the specimen was then observed under UDFM.

### Statistical analysis

All quantitative results were obtained in triplicate and expressed as the mean value. Statistical differences between evaluators A and B were analyzed using an independent-samples t-test, after confirming normal distribution and homogeneity of variance. P-value > 0.05 was considered not statistically significant.

## Results

### UDFM accurately reflects the true morphology of cartilage surface damage

**Fig. 1:**
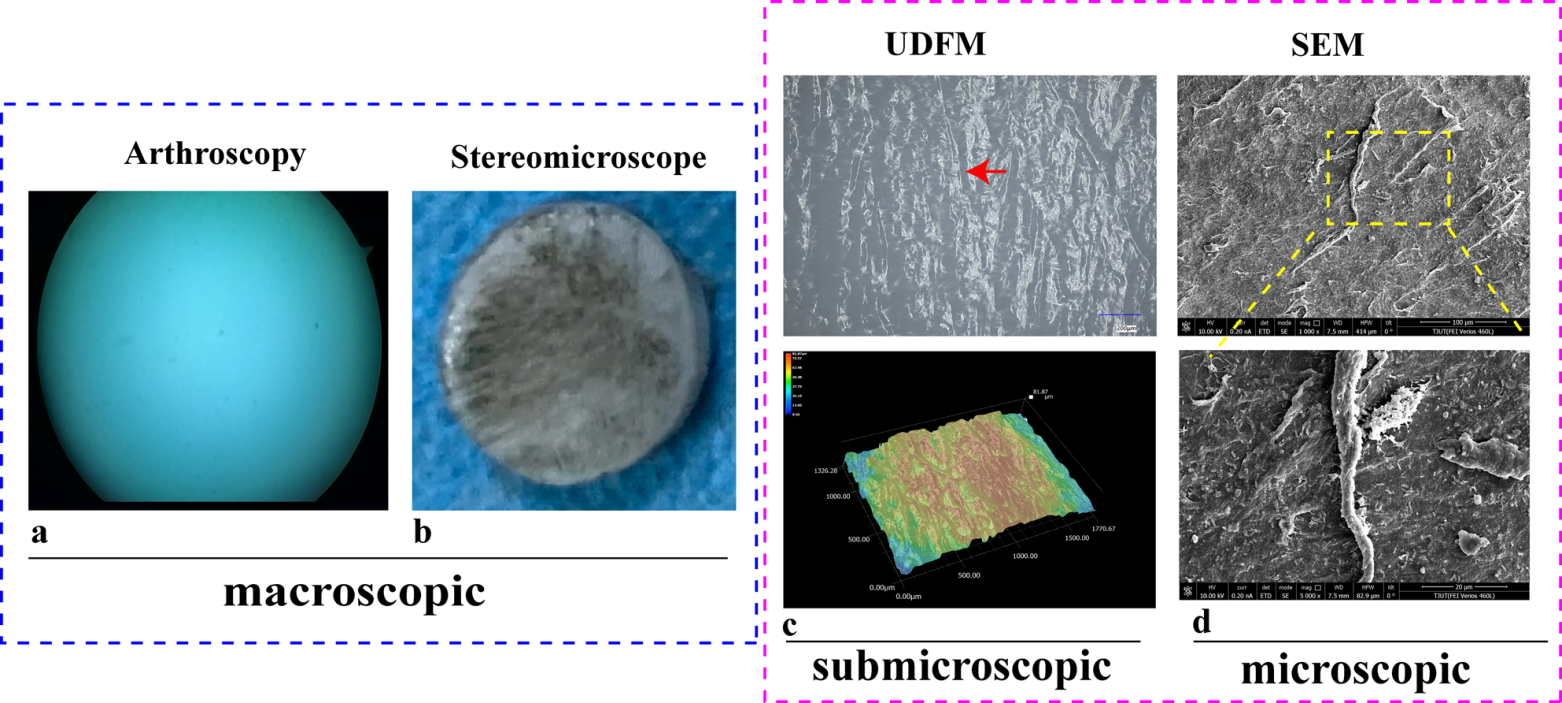
Comparison of different cartilage surface observation methods for detecting damaged cartilage. (**a**) Arthroscopic observation; (**b**) India ink staining stereomicroscope observation.Both a and b are macroscopic observation methods. (**c**) UDFM original image and three-dimensional topography, which are sub-microscopic observation methods; (**d**) SEM observation, which is a microscopic observation method

Since the characteristics of the cartilage surface are critical indicators of cartilage injury, this study used fine sandpaper to create mild surface damage and then observed the features using various surface imaging methods. Due to the subtle and superficial nature of the damage, no obvious injury marks were observed under arthroscopy (Fig. 1a). India ink staining revealed abrasion marks from the sandpaper on the surface, but due to limited magnification, the details of the damage could not be visualized (Fig. 1b). Despite its ultra-high magnification, SEM requires low-temperature gold coating and other preparatory procedures that can alter the morphology of the damage, leading to surface shrinkage(Fig. 1d). In contrast, UDFM, with its extended depth of field, was able to cover the damaged area, clearly revealing distinct linear injury marks in the images, and the three-dimensional topographic maps reflected the subtle depth and extent of the damage (Fig. 1c).

### UDFM provides distinct information at different magnifications

**Fig. 2:**
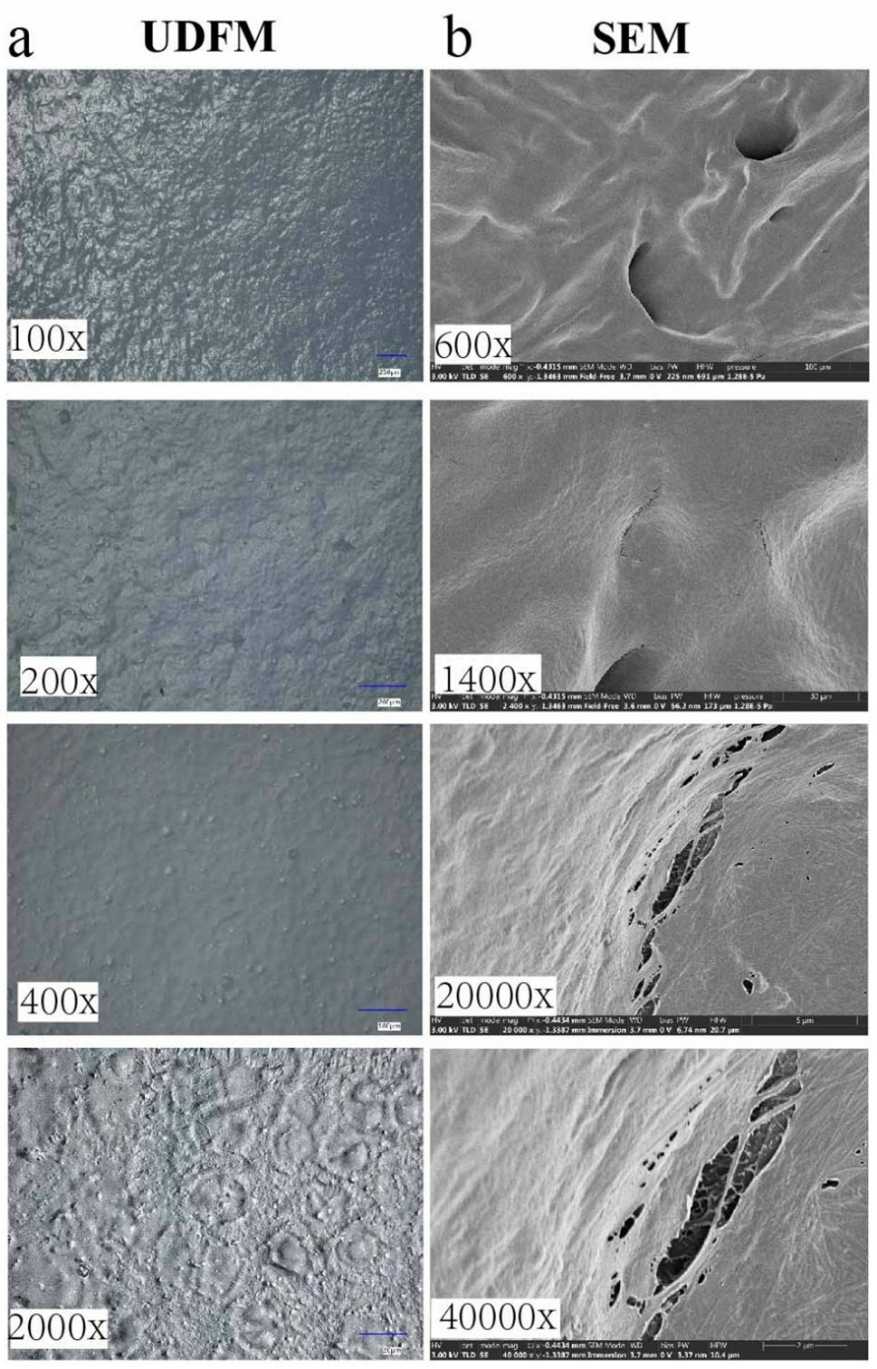
Comparison of UDFM at different magnifications. UDFM can reflect the true state of cartilage. Due to the complex sample preparation required for SEM, the original appearance of cartilage is often obscured. At 100x magnification, the overall surface morphology of cartilage appears uneven. At 200-400x magnification, the surface morphology of the matrix and its undulating state can be observed. At 2000x magnification, the morphological structure of chondrocytes is visible

UDFM offers a magnification range of 10–2000×, meeting the diverse needs of cartilage research and serving as a bridge between macroscopic gross observation and microscopic structural analysis. Observing the cartilage surface at different magnifications yields different types of information. At 100× magnification, the focus is on the macroscopic morphology, where healthy cartilage appears uneven with “granular” protrusions on the surface, yet overall displays a uniform topography without signs of friction, impact, or inflammatory damage. As the magnification increases to 2000×, paired structures resembling chondrocyte lacunae become visible on the surface (Fig. 2a). In contrast, traditional scanning electron microscopy, due to the external coating applied to the specimen, presents a much smoother and flatter surface appearance (Fig. 2b). Because UDFM does not require extensive specimen pretreatment, it more accurately preserves and reveals the true morphology of the cartilage surface, which is not perfectly smooth but rather exhibits a “granular” texture.

### Specialized OPt-SEM imaging enables precise detection of subtle cartilage surface damage

**Fig. 3:**
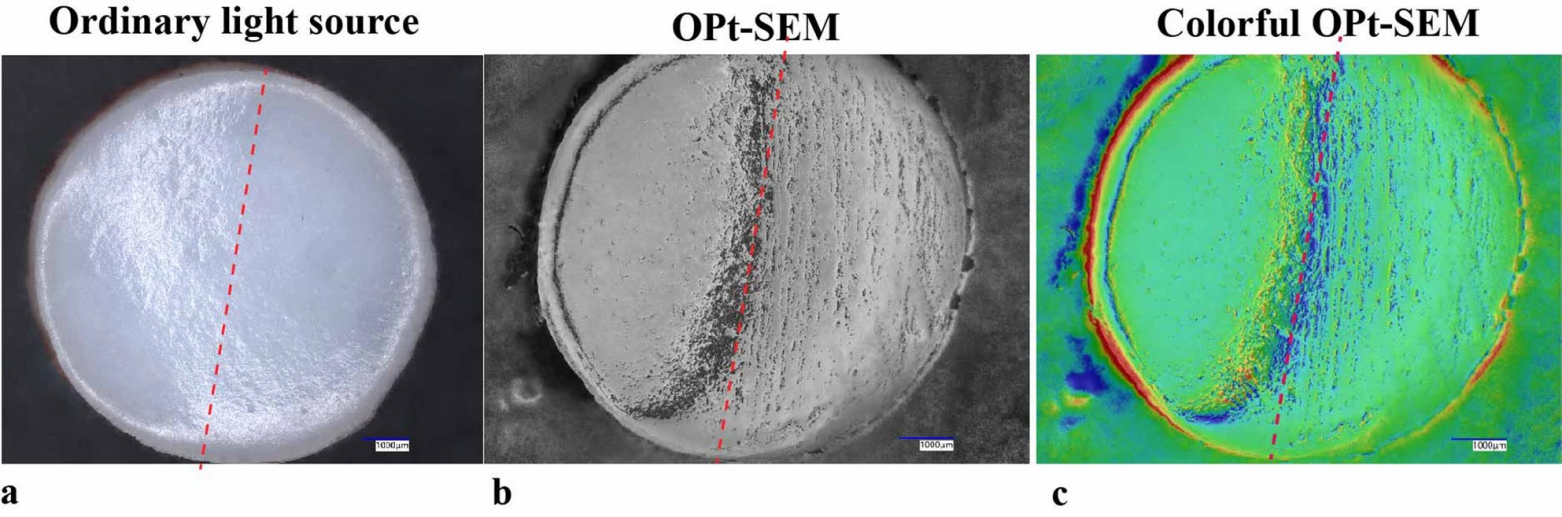
The special imaging mode OPt-SEM of UDFM is used to capture the surface of cartilage injury. (**a**) Under normal illumination mode, surface scratch marks are not obvious;. (**b**) Under OPt-SEM mode, linear scratch marks in the same area are clearly visible due to enhanced contrast from lateral illumination. (**c**) Color OPt-SEM image

The UDFM uses an LED light source that can fully illuminate the specimen. Because the cartilage surface is relatively smooth and uniformly light in color, it reflects light strongly, which may cause subtle damage to be overlooked under standard illumination. The built-in OPt-SEM mode of UDFM allows for adjustment of the light source mode and imaging method, thereby enhancing the detection of surface damage. When cartilage is abraded with sandpaper, subtle damage may not be visible under normal lighting due to the fine nature of the injury and strong surface reflection (Fig. 3a). However, when the light source is switched to OPt-SEM mode, the change in illumination direction makes abrasion marks on the surface clearly visible, creating a sharp contrast between damaged and undamaged areas (Fig. 3b, c).

### UDFM observation of cartilage lateral profiles

**Fig. 4:**
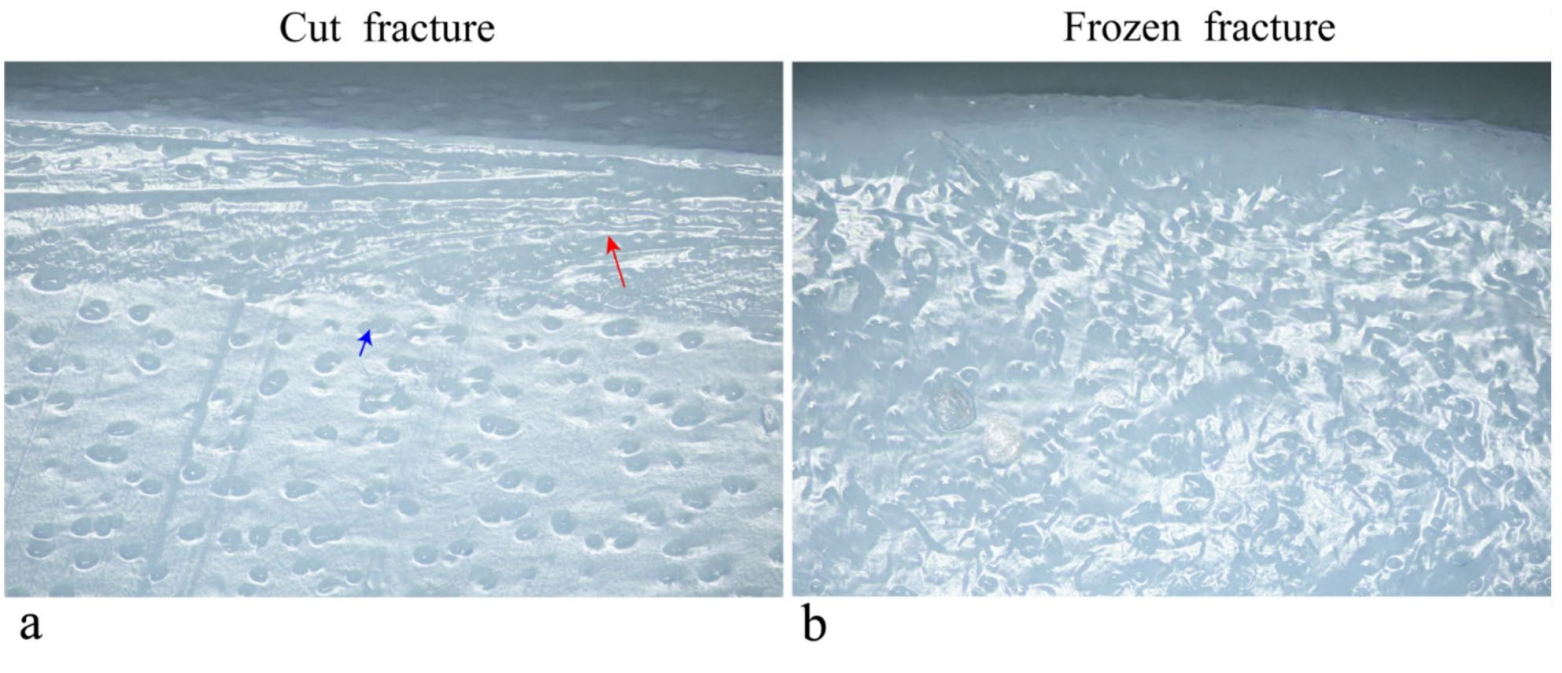
Appearance of the cartilage lateral surface after treatment by two different methods as captured by UDFM. (**a**) Lateral appearance of cartilage after vibratome sectioning; the red arrow indicates the orientation of collagen fibers, and the blue arrow indicates chondrocytes. (**b**) Lateral appearance of cartilage after liquid nitrogen freezing and fracturing

To visualize the lateral profile of cartilage and assess its structural layers, this study compared two preparation methods: vibratome sectioning and liquid nitrogen freeze-fracture. The results showed that with vibratome sectioning, UDFM could clearly reveal the orientation of collagen fibers and the structure of chondrocytes in the cartilage section (Fig. 4a). In contrast, cartilage sections prepared by liquid nitrogen freeze-fracture appeared more blurred and structurally inferior; although chondrocyte structures could be faintly observed, the overall architecture was disorganized and specific structural features were difficult to distinguish (Fig. 4b).

### UDFM accurately reflects the true morphology of chondrocytes

**Fig. 5:**
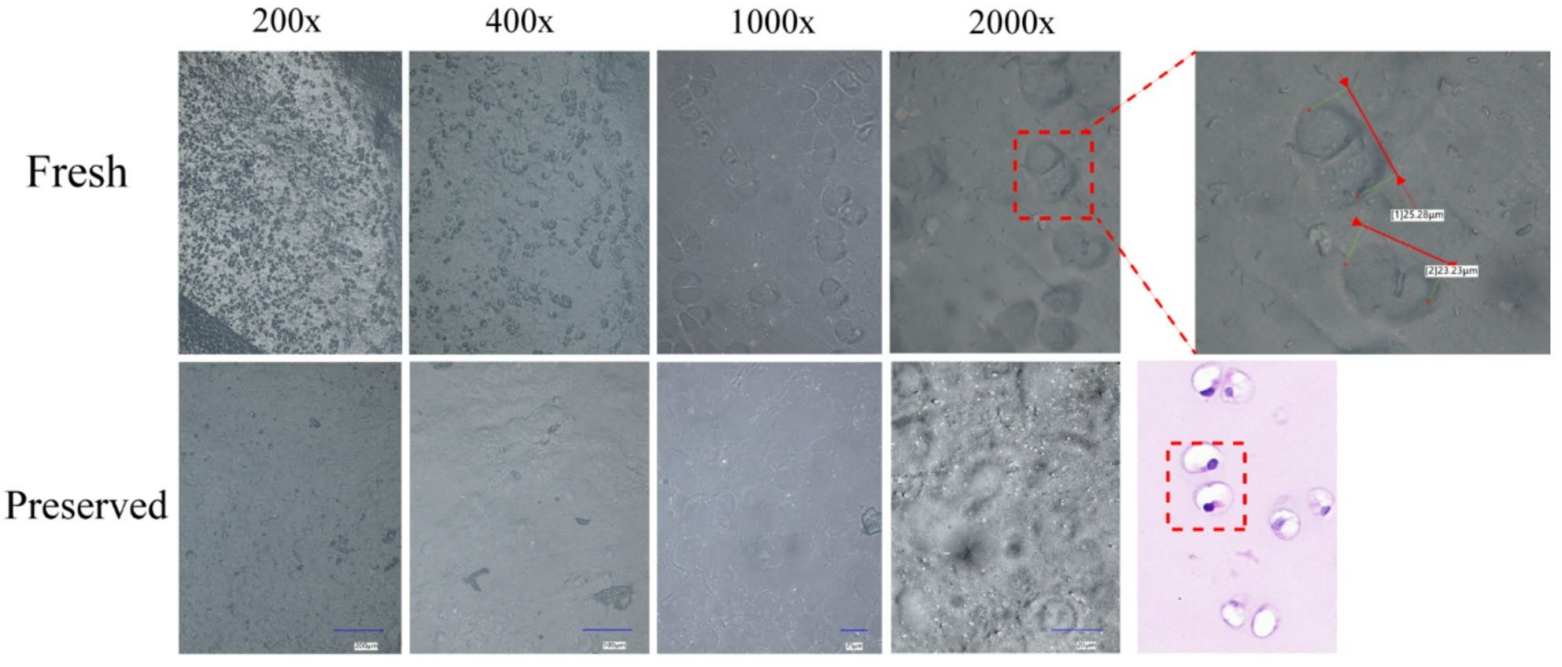
UDFM the cellular status was observed at varying degrees of freshness. In the fresh group, intact cellular structures were clearly visible, closely resembling those seen in histological staining.The diameter of the chondrocyte lacunae was measured to be 25.28 μm and 23.23 μm.In contrast, cartilage tissue preserved for more than one day rarely exhibited intact cellular structures

As previously described (Fig. 2), paired structures resembling chondrocytes were observed at 2000× magnification. Therefore, this study further examined the appearance of chondrocytes under different magnifications and conditions. At higher magnification, the chondrocyte structures closely resembled those seen in histological staining, appearing in pairs or clusters. These chondrocytes represent the native, unaltered state of cells on the cartilage surface and can only be observed in fresh cartilage specimens. In non-fresh samples or those left ex vivo for more than eight hours, the structures appeared blurred, cell contours collapsed, and in some cases, chondrocyte structures could not be visualized at all. These results indicate that UDFM enables the most direct and immediate observation of chondrocyte features (Fig. 5). This method does not require any pretreatment of the specimen prior to observation and allows for visualization of intact cellular morphology, making it a rapid, convenient, and intuitive approach.

### The UDFM can identify regions of different colors and measure the area of captured colors

**Fig. 6:**
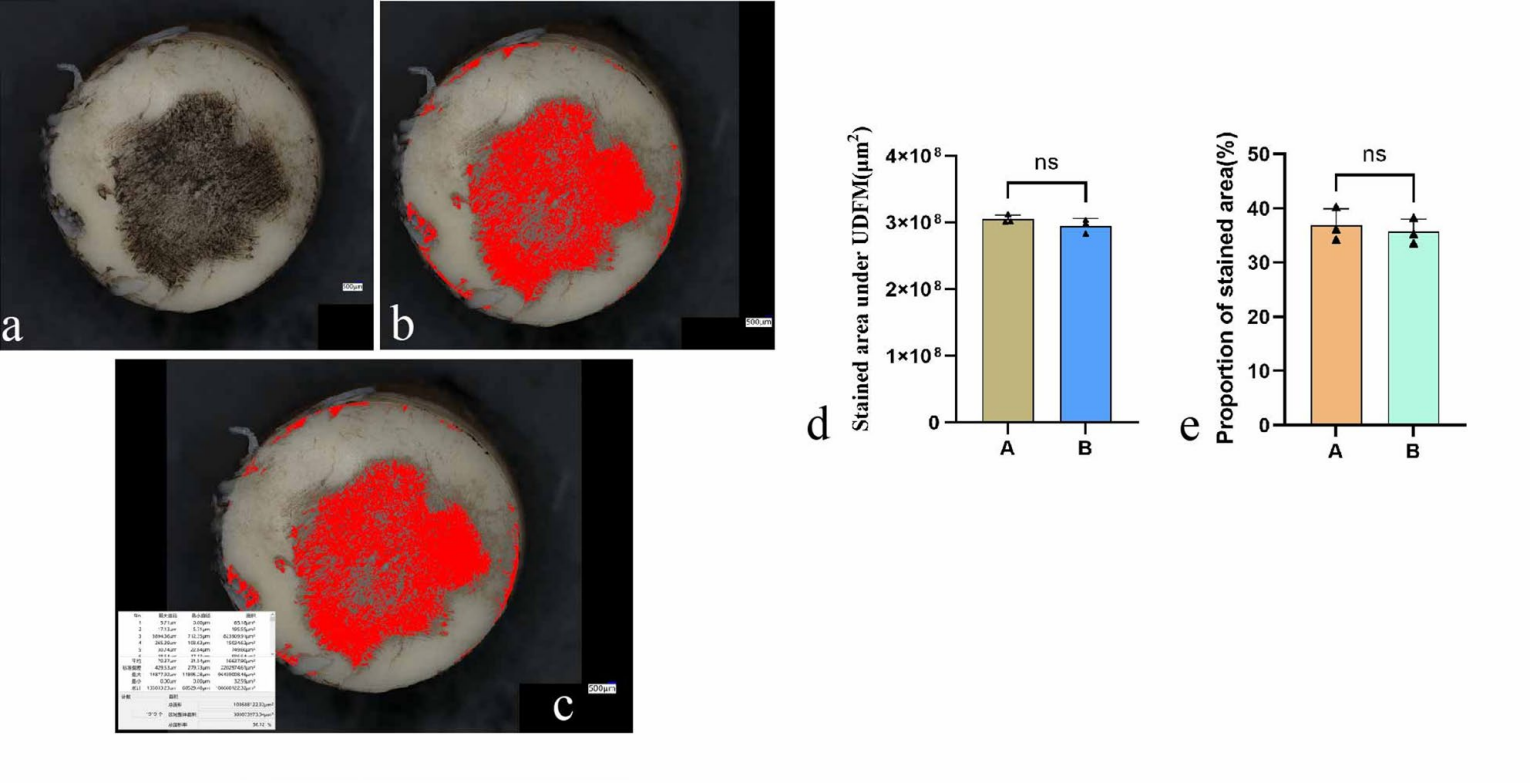
UDFM analysis of differently colored regions and their proportional areas on the cartilage surface. (**a**) UDFM image at 50× magnification following India ink staining. (**b**) Identification of black-stained injured regions. (**c**) Calculation of the absolute area and relative proportion of black-stained regions relative to the total cartilage area. (**d**) Quantification of the stained area and comparison between measurements obtained by method A and method B. (**e**) Quantification of the proportion of stained area relative to the total cartilage surface area and comparison between measurements obtained by method A and method B.Data were analyzed using independent-samples t-test, *n* = 3,*p* > 0.05

The UDFM can capture the black regions of India ink staining on the cartilage surface and automatically detect all black areas and their proportions within the designated region. After marking the damaged cartilage surface with India ink, the injured area appears black (Fig. 6a). All black regions are captured and highlighted (Fig. 6b). The area of cartilage injury, i.e., the black area, was calculated to be 108,688,122.32 μm², while the total selected area was 300,873,973.34 μm², accounting for 36.12% (Fig. 6c). There was no statistically significant difference in the measured stained area and proportion between observers A and B (Fig. 6d, e).

### Accurate measurement of cartilage height differences by UDFM

**Fig. 7:**
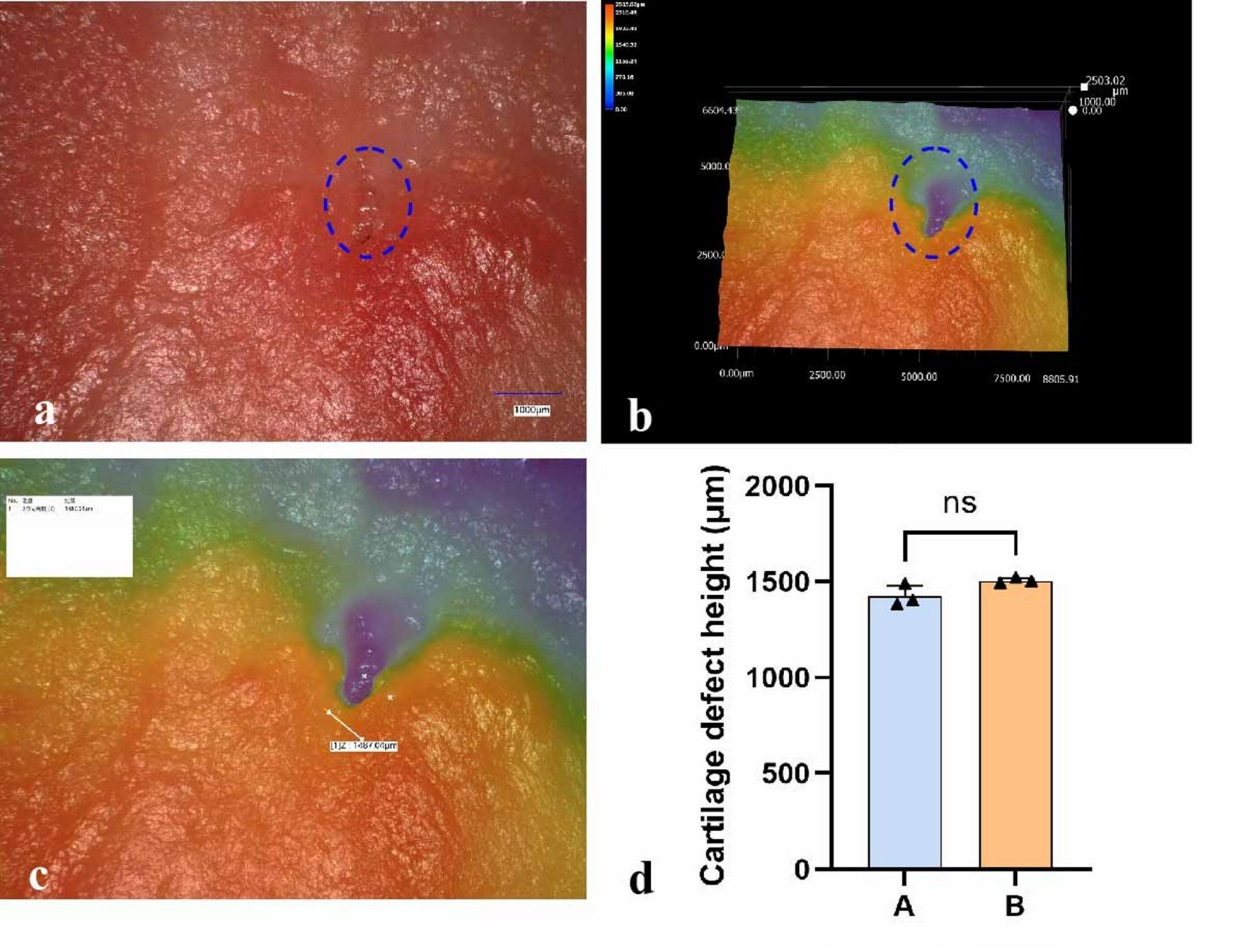
Measurement of height difference in the region by UDFM. (**a**) The original image of the cartilage surface is captured, with the blue area indicating the cartilage defect region. (**b**) Three-dimensional topography reconstruction shows that the defect region appears as a depressed blue area. (**c**) The height difference between the center of the defect and the surrounding cartilage is measured in the 3D topography map. (**d**) Quantification of cartilage defect height and the difference between measurements by A and B.Data were analyzed using independent-samples t-test, *n* = 3,*p* > 0.05

In cartilage research, it is often necessary to perform real-time measurements of observed cartilage defects to determine defect depth. However, conventional measurement methods are often not convenient or simple for real-time assessment and usually require additional data processing. UDFM, by performing multi-layer continuous scanning of the specimen surface, can obtain complete positional information of all surface points.To evaluate the effectiveness of UDFM for this indicator, discarded cartilage from human TKA procedures was observed under the UDFM in this study. The original images showed a central cartilage defect (Fig. 7a, b). When scanning to the defect site, the software’s measurement function allows for accurate measurement on both the original image and the 3D topography. The height difference can be measured directly on the generated 3D topographic map (Fig. 7c), and the cartilage defect was found to be 1,487.04 μm. Repeated measurements by observers A and B showed no significant difference (Fig. 7d). This intuitive and convenient measurement method can greatly satisfy the needs of cartilage-related research.

### Measurement of cartilage surface roughness by UDFM to reflect surface topography

**Fig. 8:**
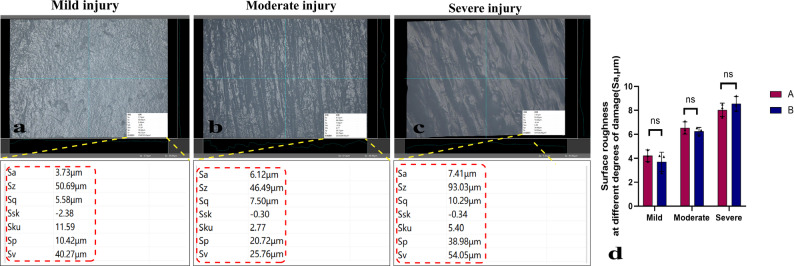
UDFM measurement of cartilage surface roughness and area after sandpaper friction. (**a**) Surface of mildly abraded cartilage. (**b**) Surface of moderately abraded cartilage. (**c**) Surface of severely abraded cartilage. The red dashed line indicates the roughness value. (**d**) Quantified roughness values at varying injury severities and the difference between measurements by A and B. Data were analyzed using independent-samples t-test, *n* = 3, *p* > 0.05

As the primary site of interfacial friction, the surface roughness of cartilage is an important mechanical property and is crucial for cartilage research. In this study, sandpaper friction was applied to create three different cartilage injury models—mild, moderate, and severe. Observation under the UDFM revealed that the damage marks on the cartilage surface became more pronounced with increasing injury severity, manifested as deeper and coarser grooves. By selecting the entire field of view, the roughness values for mild, moderate, and severe injuries were found to be 3.73 μm, 6.12 μm, and 7.41 μm, respectively, indicating that surface roughness increased with the degree of injury (Fig. 8a, b, c). Repeated measurements by observers A and B showed no significant difference (Fig. 8d). This convenient method enables rapid and accurate acquisition of mechanical information, enriching cartilage-related research.

### Measurement of cartilage surface fissure length by UDFM

**Fig. 9:**
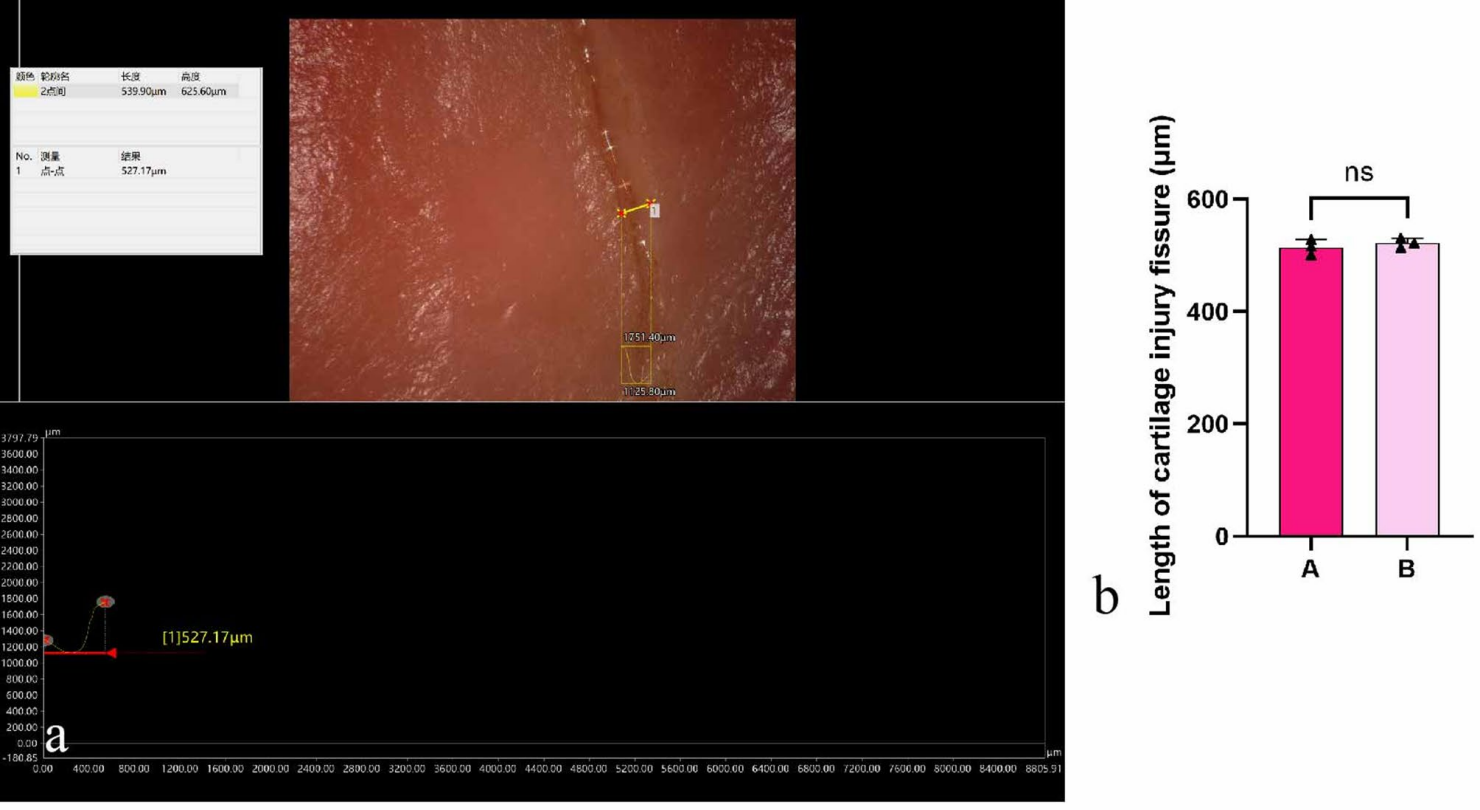
UDFM measurement of cartilage surface fissure length. (**a**)The fissure length region is delineated, and a length profile of this region appears below the image. (**b**)Quantification of cartilage injury fissure length and the difference between measurements by A and B.Data were analyzed using independent-samples t-test, *n* = 3, *p* > 0.05

In cartilage research, it is often necessary to measure observed injury sites. In this study, cartilage tissue excised after TKA was observed under the UDFM, revealing a fissure in the cartilage. The fissure width was measured directly from the original image, with the value displayed at the bottom of the interface; the fissure width was 527.17 μm (Fig. 9a). Repeated measurements by observers A and B showed no significant difference (Fig. 9b). This precise measurement of subtle injuries meets the refined requirements of cartilage research.

## Discussion

Although UDFM has been introduced as a novel technical approach, its true transformative potential lies in the unique functional insights it provides into cartilage physiology that are inaccessible via conventional modalities. Unlike stereomicroscopy, SEM, and confocal microscopy, which excel at morphological rendering, UDFM quantifies surface frictional characteristics—key biomechanical biomarker that precede visible structural damage.

Conventional imaging techniques, including stereomicroscopy, confocal microscopy, SEM, and OCT, primarily focus on the characterization of morphology and structure [15]. In contrast, UDFM enables the integrated analysis of both morphological structure and biomechanical properties within a single assessment, which cannot be achieved by these established methods.Meanwhile UDFM offers wide-field, label-free visualization of superficial surface integrity at high speed, eliminating the need for exogenous labels and enabling real-time assessment. This capability positions UDFM as a critical translational tool, bridging the gap between ex vivo structural analysis and in vivo functional monitoring. By capturing these uncharted functional dimensions, UDFM opens new avenues for the early diagnosis and mechanistic study of cartilage degeneration.

Traditional macroscopic approaches for cartilage surface evaluation, including arthroscopy [16], India ink staining, and stereomicroscopy, are limited by low magnification, staining requirements, and low sensitivity to subtle surface damage. Conversely, microscopic methods such as HE staining and SEM involve complex, invasive sample preparation that may alter the native tissue state. UDFM functions as a sub-microscopic imaging technique with a magnification range between stereomicroscopy and SEM. It features simple operation, minimal sample intervention, and reliable quantitative capability, making it highly suitable for characterizing cartilage surface morphology (Supplementary Fig. 4).

UDFM enables intuitive three-dimensional visualization of key cartilage surface microfeatures, including undulations, fissures, wear, and fiber alignment. This 3D reconstruction supports accurate interpretation of the native cartilage surface and overcomes the limitations of conventional two-dimensional imaging.

Magnification is a critical parameter in UDFM imaging. At below 100×, UDFM provides an overview of macroscopic features, including general contours, large-scale defects, and surface wear. Between 100× and 400×, it resolves detailed structures such as chondrocyte distribution, matrix organization, and early degenerative changes. Above 400×, it enables high-resolution analysis of chondrocyte morphology and microscale pathological alterations including degeneration, apoptosis, and matrix degradation.

It is noteworthy that during UDFM observation of fresh cartilage tissue, pairs of chondrocytes arranged in close proximity—two cells tightly adjacent to each other with highly consistent morphology, size, and orientation—could be directly visualized. This phenomenon closely resembles findings from conventional histological staining, supporting the capability of UDFM to reflect the true state of chondrocytes. Notably, in situ observation of paired chondrocytes in their native state has been rarely reported in previous studies. Traditional methods often involve sectioning, dehydration, and embedding, which readily disrupt the native spatial relationships between cells. In contrast, UDFM enables more authentic visualization of chondrocyte distribution and interactions within their natural microenvironment, providing a new tool for investigating chondrocyte proliferation, migration, and differentiation. In situ observation of paired chondrocytes also helps to illustrate dynamic cellular changes and functional specialization.

The unique qualitative and quantitative structural analysis capabilities of UDFM provide clear advantages for cartilage surface research. Its non-destructive, efficient three-dimensional imaging achieves depth of field and resolution that are difficult to obtain with conventional optical microscopy, making it highly suitable for evaluating complex cartilage surface structures.

Quantitative UDFM assessment allows comprehensive characterization of cartilage surface features. Height variation reflects the severity of surface defects and serves as a key indicator for evaluating integrity, smoothness, and microdamage. In the present study, observation of clinical TKA specimens revealed obvious local height differences, indicating surface wear or fissures, thus providing valuable diagnostic information. UDFM enables micron-level quantitative topographic assessment without complex sample preparation, avoiding damage associated with mechanical profilometry or SEM and allowing real-time evaluation of native cartilage.

Surface roughness is a key index reflecting cartilage micro-irregularity; increased roughness generally indicates matrix degradation, fiber exposure, or impaired repair, and represents an important marker of cartilage degeneration and injury [17–19]. In our damage models, UDFM allowed rapid, non-destructive acquisition of three-dimensional data and accurate calculation of roughness parameters. Compared with conventional contact profilometry or SEM, UDFM is more efficient and less invasive. Our results showed that surface roughness increased gradually with damage severity, which may further influence cartilage biomechanical properties and function [20]. Notably, roughness values increase linearly with magnification [21]; thus, consistent magnification is required for comparative analysis. Quantitative roughness assessment shows potential for cartilage grading and repair evaluation [19], offering an objective basis for clinical evaluation.

Three-dimensional distance measurement represents another important quantitative approach for cartilage surface microstructure analysis. Accurate measurement of fissure width, fiber bundle length, and defect dimensions allows direct evaluation of injury severity, degeneration, or repair status. In this study, UDFM enabled high-precision three-dimensional length measurement of micro-fissures, expanding the toolkit for cartilage injury assessment. By overcoming the limitations of traditional two-dimensional imaging, UDFM provides a robust approach for the objective evaluation of cartilage injury and repair.

Despite the promising performance of UDFM for characterizing cartilage surface properties, the present study was limited to ex vivo specimens rather than clinical disease or cartilage repair models. Therefore, the current findings should be primarily interpreted as a methodological validation of UDFM for quantitative cartilage evaluation, and the claims regarding early diagnosis and repair assessment have been appropriately moderated.

Nevertheless, the present study also acknowledges several limitations of UDFM.First, the imaging depth of UDFM is relatively limited, and it is mainly suitable for the observation of cartilage surface and superficial layers, rather than deep internal structures.Second, UDFM is sensitive to liquid residues on the sample surface, which may interfere with light reflection and thus affect imaging clarity.Third, although UDFM provides clear morphological visualization of cartilage surface, it cannot achieve ultra-high-resolution imaging at the subcellular organelle level and therefore cannot replace electron microscopy for ultrastructural observation.These limitations suggest directions for further technical optimization and application expansion of UDFM in future studies.

Future translation of UDFM may focus on integrating this technique into clinical and translational workflows, such as intraoperative evaluation of cartilage degeneration during arthroscopy, longitudinal monitoring of repair tissue after surgery, and quality assessment of tissue-engineered cartilage before implantation. By enabling simultaneous visualization of structural morphology and mechanical properties, UDFM holds potential to provide objective, quantitative indicators that complement conventional clinical imaging and histological evaluation, thereby facilitating more accurate diagnosis and personalized assessment of cartilage repair in clinical practice.

## Conclusion

This study systematically evaluated the application value of the UDFM in characterizing cartilage surfaces. The results demonstrate that UDFM enables non-destructive and intuitive acquisition of three-dimensional topographical information of the cartilage surface, allowing for precise quantitative and qualitative measurement of multiple parameters, including surface damage area, height difference, roughness, and inter-point distance. This technique offers significant advantages in terms of resolution, depth of field, operational convenience, and preservation of native structure, providing a more comprehensive and accurate reflection of the microscopic structure and damage characteristics of the cartilage surface. UDFM is not only suitable for research related to cartilage surfaces, but also provides a novel tool for the objective evaluation of repair outcomes such as cartilage transplantation. In summary, UDFM opens a new technical avenue for both basic and clinical research on cartilage surfaces and holds broad application prospects.

## Supplementary Information

Below is the link to the electronic supplementary material.

Supplementary Material 1

Supplementary Material 2

## Data Availability

The data are available from the corresponding author on reasonable request.
