# Supplementary material for: Ultra depth of field microscopy: a novel method for observing and characterizing of articular cartilage surface
Source: J Transl Med. 2026 Mar 25;24:627. doi: 10.1186/s12967-026-08060-x (PMC13141283; doi:10.1186/s12967-026-08060-x)
Supplement: Supplementary file 1 — Supplementary Material 1 [file 12967_2026_8060_MOESM1_ESM.docx]

**Supplementary Figures**

**Supplementary Figure 1.Schematic diagram of each part of the UDFM. It consists of five parts: (a) Main display screen of the ultra-depth-of-field microscope, (b) Electromechanical unit, (c) Optical magnifying lens, (d) Digital camera lens, and (e) Control handle.**

**Supplementary Figure 2.UDFM imaging process: the information of the target object is recorded as digital signals in the device storage, and the original spatial information of the target object is reconstructed through decoding.**

**
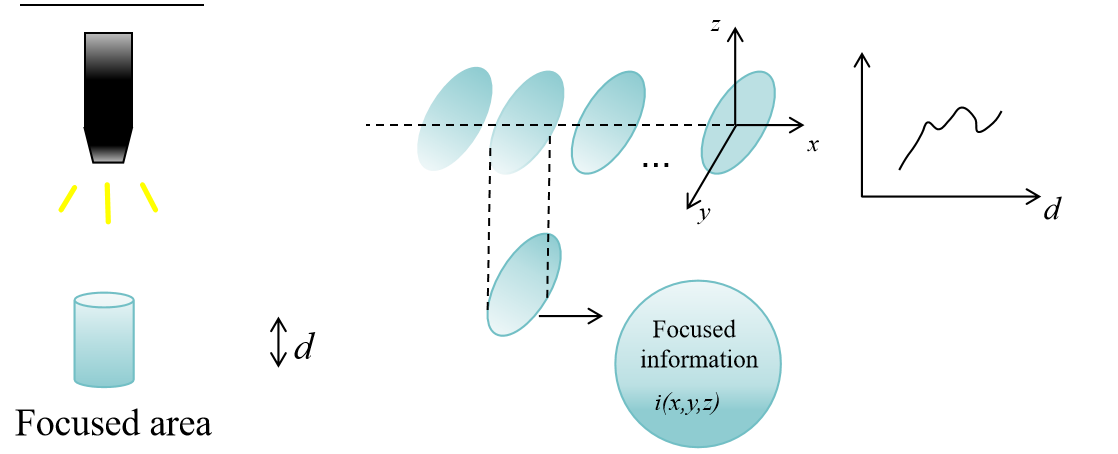
**

**Supplementary Figure 3.UDFM imaging principle: The object is scanned at different layers, and the positional information from the scan is stored in the internal recording device via an internal signal encoder. The original scanned object signals are then decoded according to the recorded layer information, and images are generated.**

**
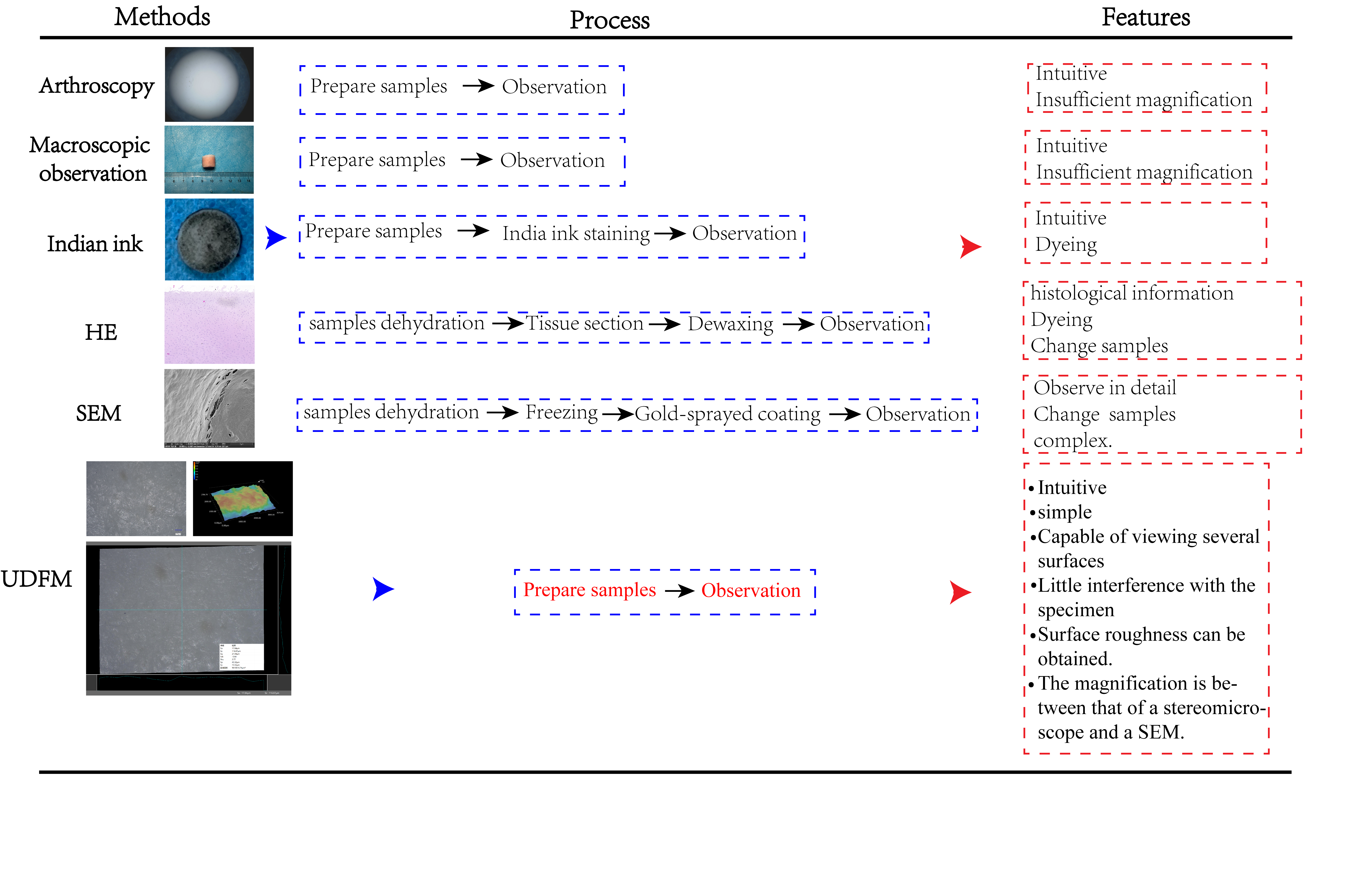
Supplementary Figure 4.Advantages of UDFM over other observation methods: UDFM features a simple process, is easy to operate, and is friendly to specimens. It does not damage the original morphology of cartilage and greatly preserves the surface characteristics of the cartilage.**
